# An Instantaneous Recombination Rate Method for the Analysis of Interband Recombination Processes in ZnO Crystals

**DOI:** 10.3390/ma15041515

**Published:** 2022-02-17

**Authors:** Luigi Santamaria, Pasqualino Maddalena, Stefano Lettieri

**Affiliations:** 1 Italian Space Agency (ASI), Space Geodesy Center “G. Colombo”, 75100 Matera, Italy; luigi.santamaria@asi.it; 2 Dipartimento di Fisica “E. Pancini”, Università degli Studi di Napoli “Federico II“, Complesso Universitario di Monte S. Angelo, Via Cupa Cintia 21, 80126 Napoli, Italy; pasqualino.maddalena@unina.it; 3 Istituto di Scienze Applicate e Sistemi Intelligenti “E. Caianiello”, Consiglio Nazionale delle Ricerche (CNR-ISASI), Complesso Universitario di Monte S. Angelo, Via Cupa Cintia 21, 80126 Napoli, Italy

**Keywords:** photoluminescence, charge carriers, recombination, zinc oxide, excitons, time-resolved photoluminescence, kinetics

## Abstract

Time-resolved photoluminescence (TRPL) analysis is often performed to assess the qualitative features of semiconductor crystals using predetermined functions (e.g., double- or multi-exponentials) to fit the decays of PL intensity. However, in many cases—including the notable case of interband PL in direct gap semiconductors—this approach just provides phenomenological parameters and not fundamental physical quantities. In the present work, we highlight that within a properly chosen range of laser excitation, the TRPL of zinc oxide (ZnO) bulk crystals can be described with excellent precision with second-order kinetics for the total recombination rate. We show that this allows us to define an original method for data analysis, based on evaluating the “instantaneous” recombination rate that drives the initial slope of the decay curves, acquired as a function of the excitation laser fluence. The method is used to fit experimental data, determining useful information on fundamental quantities that appear in the second-order recombination rate, namely the PL (unimolecular) lifetime, the bimolecular recombination coefficient, the non-radiative lifetime and the equilibrium free-carrier concentration. Results reasonably close to those typically obtained in direct gap semiconductors are extracted. The method may represent a useful tool for gaining insight into the recombination processes of a charge carrier in ZnO, and for obtaining quantitative information on ZnO excitonic dynamics.

## 1. Introduction

The phenomenon of band-to-band (or “interband”) photoluminescence (PL) in semiconductor materials is caused by the radiative recombination of free charge carriers (i.e., valence holes and conduction electrons), generated via the absorption of optical radiation, whose photon energy is larger than the bandgap energy of the material.

Surface traps, bulk defects and band offsets at interfaces significantly affect the rates of charge recombination [1,2,3,4] and thus of PL intensity, by introducing efficient non-radiative recombination pathways and/or by affecting the average spatial separation between electrons and holes. As a consequence, the optical PL spectroscopy of semiconductors is often employed to assess crystal quality and surface/interface characteristics [5,6,7,8,9,10]. Very often, these assessments do not involve quantitative analysis and phenomenological approaches are sufficient for extracting the desired information. A typical example of that is the use of a predetermined decaying function to fit the time-resolved photoluminescence (TRPL) decays. For example, lifetimes are often extracted by fitting the data with a sum of exponentially-decaying functions. However, such lifetimes are just phenomenological parameters and not fundamental physical quantities. In fact, a multi-exponential function would be the correct description of a PL decay only if the total recombination was caused by different populations of excited charges all decaying simultaneously via a first-order kinetic process. In reality, this does not occur: it is instead well established in the theory of interband recombination [11,12] that the electron-hole decay does not follow first-order kinetics, except for very low charge densities. Moreover, there is just one population of excited charges involved in a direct-bandgap semiconductor, namely the free carrier population (electrons energetically close to the minimum of the conduction band and holes close to the maximum of the valence band).

Performing quantitative analyses that involve the actual physical parameters can be especially relevant for gaining insights on the charge-carrier recombination mechanisms. This is particularly valid in regard to semiconductors relevant for their light-emitting and/or photocatalytic and/or gas-sensing properties. The validity of this statement is quite evident in regard to the light-emitting properties, as the efficiency of interband PL is a prerequisite for achieving efficient amplified spontaneous emission and lasing [13].

In regard to photocatalytic properties, the key point is that a photocatalyst provides free charges on the semiconductor surface for chemical redox reactions of adsorbed molecules. Once used for a reduction or an oxidation reaction, the free charge is no longer available for PL emission: therefore, PL and photocatalytic processes are in competition. For this reason, monitoring of the PL intensity is often employed to probe photocatalytic efficiency and to monitor surface reactions [14,15,16,17].

Additionally, the photo-generation of excited charges always occurs in a region close to the sample surface. Hence, changes in the gaseous environment surrounding the sample typically determine changes in the charge recombination probability, i.e., in the PL intensity and lifetime. For this reason, PL spectroscopy is a sensitive technique for analyzing surfaces [18], and optically-based gas sensing can be achieved by monitoring the PL lifetime and/or intensity modulation [19,20,21].

Zinc oxide (ZnO) is a metal oxide semiconductor that exhibits all the three mentioned functionalities. It is an efficient light emitter [22,23] thanks to its direct bandgap. It is also a well-known photocatalyst, relevant in the field of visible light-activated photocatalysis (also known as “solar” photocatalysis) and of photo-induced antibacterial surface functionalization [24,25,26]. Finally, ZnO is a gas-sensitive semiconductor employable as both a conductometric [27,28,29] and optical [30,31,32] sensor.

Based on these considerations, we focused the present work on developing a simple method for the data analysis of TRPL in ZnO, aiming to determine the actual physical quantities that are defined in the second-order rate equation for the electron-hole recombination, namely, the unimolecular lifetime, τ, the bimolecular coefficient, B and the equilibrium density of free carriers, N. In order to simplify the exposure, the definitions and physical meaning of these quantities are discussed in more detail in Appendix A.

In order to achieve the aim of this work, we firstly highlighted that the interband recombination in ZnO subjected to pulsed laser excitation at moderate values of optical fluences, is described with good precision by a total electron-hole rate of recombination of the second order (i.e., a second-order kinetic process). This is here clearly evidenced experimentally by a transition from an almost single-exponential decay at very low optical fluences, to a bimolecular recombination decay at moderate optical fluences. These different decay kinetics affect mainly the initial stage of the PL decay; this allowed us to focus on the initial slope of the PL decay curves and to define a procedure, based on the initial “instantaneous lifetime”, that can provide a quantitative determination of the above-mentioned characterizing parameters.

The procedure is described in the next section, followed by the discussion and conclusions.

## 2. Materials and Methods

Measurements of continuous-wave, time-resolved and time-integrated photoluminescence—labelled as CWPL, TRPL and TIPL, respectively—were carried out at room temperature on (0001) oriented single crystal ZnO substrates purchased from CrysTec GmbH (hydrothermal growth, 10 × 5 mm^2^ surface area, 0.5 mm thickness). Two as-received crystals belonging to the same batch were characterized by means of TRPL, TIPL and continuous-wave photoluminescence (CWPL). Laser excitation for CWPL measurements was provided by the 325 nm wavelength of an He-Cd laser (10 mW, laser spot size of approximately 4*10^−2^ cm^2^). Pulsed laser excitation for TRPL was provided by the third harmonic beam of a mode-locked Nd:YAG laser, characterized by the following parameters: 355 nm wavelength, 25 picoseconds pulse duration (average full width at half the maximum of the temporal profile of the laser pulses) and 10 Hz repetition rate. TIPL spectra $\Phi \left(\lambda \right)$ were determined by the time integration of the TRPL intensities $\phi \left(t\right)$, i.e.,:

(1) $$\Phi \left(\lambda \right)=\int_{\Delta t}\phi \left(t,\lambda \right)dt$$

where Δt = 2 ns is the time range of over which the TRPL spectra were acquired.

The excitation laser beam was slightly focused on the sample surface, impinging with about a 50° angle of incidence and a 10^−1^ cm^2^ laser spot area. The sample’s reflectivity (R) for different values of laser excitation fluency was obtained by measuring the energy of impinging and reflected laser pulses by means of a pyroelectric joulemeter. A wedge window placed along the laser path was used to extract a fraction of excitation energy and to direct it towards an energy meter in order to monitor the laser energy.

The photoluminescence emission emerging from the excited surface was collected by means of an achromatic confocal lens system. The time-resolved PL spectral components were dispersed by a spectrometer (25 cm equivalent focal length) and detected via a streak camera optically coupled with the exit slit of the spectrometer. The acquisition electronics of the streak camera were externally triggered by the laser, allowing shot-by-shot detection. The overall system provided simultaneous time-resolved and wavelength-resolved detection (overall experimental time and spectral resolution: 30 ps and 1 nm, respectively). The residual signal at excitation wavelength (355 nm) was eliminated by means of a sharp cut-off optical filter.

For each sample, multiple TRPL data were acquired using different values of the optical excitation fluency, F, defined as the laser pulse energy absorbed by the crystal, divided by the laser spot area at sample surface. It is worth underlining that the large thickness of the crystals (d = 500 μm) assures that all the samples were optically thick, i.e., αd≫1 where α is the absorption coefficient of the ZnO crystal at 355 nm wavelength. Given a typical value of α ≈ 10^5^ cm^−1^, which corresponds to an optical penetration length of 100 nm, the Lamber-Beer low assures that 99.7% of the optical energy is absorbed within a thickness of about 0.3 μm. Considering that our crystals were 500 μm thick, the complete absorption of the optical energy which is not reflected away by the sample surface is assured.

## 3. Results

The results are presented in two separate subheadings, where the first describes the theoretical model used to set the analytical procedure of data analysis, while the second shows and discusses the experimental results obtained for ZnO bulk crystals.

### 3.1. Theoretical Model

The model presented in this work considers a n-type direct gap semiconductor in which an equilibrium density of free carriers, indicated by N, is present in the conduction band even in absence of photoexcitation, due to the partial ionization of shallow donors. In addition, photoexcitation by laser pulses induces the presence of non-equilibrium (i.e., time dependent) excess concentration, indicated by $\rho \left(t\right)$. After pulsed laser excitation, the electronic fundamental state (i.e., equilibrium) is restored through the recombination of the excess carriers, whose time evolution is determined by the rate equation:

(2) $$-d\rho /dt=W\left(\rho \left(t\right)\right)$$

where the quantity W represents the total recombination rate, defined as the total number of recombination events (including both radiative and non-radiative ones) per unit time and volume. It is worth noting that the total recombination rate is itself a function of the excess charge density, as it has been explicitly indicated.

We further indicate by $W_{R}$ and $W_{NR}$ the radiative and non-radiative recombination rates, respectively, so that $W\left(\rho \left(t\right)\right)=W_{R}\left(\rho \left(t\right)\right)+W_{NR}\left(\rho \left(t\right)\right)$. Again, our nomenclature explicitly expresses the fact that both recombination rates depend on *ρ*.

As an interband electron-hole recombination occurs as a two-body scattering event, its probability is proportional to the product between the hole density p=ρ and the electron density n=N+ρ, leading to the following general expression of a radiative recombination rate [11]:

(3) $$W_{R}\left(t\right)=BN\rho \left(t\right)+B\rho^{2}\left(t\right)≝\rho \left(t\right)/\tau_{R}+B\rho^{2}\left(t\right)$$

where the coefficient *B* is the radiative bimolecular coefficient, and where the second equality defines the radiative unimolecular lifetime $1/\tau_{R}≝BN$.

The quantity $W_{R}\left(t\right)$ gives, by definition, the number of emitted (i.e., photoluminescent) photons per unit volume and time. Therefore, the experimentally measured time-resolved photoluminescence intensity $\phi \left(t\right)$ at each wavelength is proportional to the expression of $W_{R}\left(t\right)$, which is obtained by using in Equation (3) the function $\rho \left(t\right)$ that solves the rate equation Equation (2).

It is immediately seen that a recombination rate W that is linearly proportional to *ρ*, would be trivial and unrealistic, as it would lead to a single-exponential decay for both the excess charge density and the photoluminescence intensity. Such a simple case is almost never encountered in the actual experimental results for photoluminescence decay in oxides and, even more relevantly, does not describe the experimental results discussed later.

Therefore, a less simple expression is required. The core of the present model is to consider the expression of $W\left(\rho \left(t\right)\right)$ up to the second order in terms of the power of the $\rho \left(t\right)$ variable, that is to use a generalized second-order recombination rate $W^{\left(2\right)}=a\rho +b\rho^{2}$ and to solve the following rate equation:

(4) $$-\frac{d\rho }{dt}=W^{\left(2\right)}≝\frac{\rho }{\tau }+b\rho^{2}$$

where the unimolecular lifetime τ appears in the definition of the linear term (i.e., a=1/τ). The rate Equation (4) thus neglects the eventual contributions of the third order $\left(\rho^{3}\right)$ and is successive to the total recombination rate.

The radiative recombination rate in interband transition is intrinsically of second order vs. charge density, as shown in Equation (2). Hence, to discuss the approximations implied by Equation (3), we have to consider the non-radiative recombination.

The main non-radiative recombination processes in semiconductors are the Shockley-Read-Hall (SRH) recombination and the Auger recombination. The SRH processes consist of the simultaneous capture of an electron and a hole at deep defect levels, and various kind of defects (extrinsic as well as intrinsic) can give place to SRH recombination, which thus significantly limit the radiative recombination probability, unless the material has high crystalline quality. On another hand, the Auger processes are three-body processes, in which the kinetic energy of a pair is transferred to a third charge carrier. As such, an Auger process involving three free carriers is proportional to the third power (*ρ*^3^) of the injected carrier density and can hence become important at large densities of photogenerated carriers.

To summarize, the use of a second-order effective recombination rate is reasonable, as far as exceedingly high optical injection regimes are not probed experimentally. As shown in the next section, this hypothesis allows us to significantly simplify the analysis, while maintaining the main experimental features.

The solution of the second-order differential Equation (4) is:

(5) $$\rho \left(t\right)=\frac{\rho_{0}\exp\left(-t/\tau \right)}{1+b\rho_{0}\tau \left(1-\exp\left(-t/\tau \right)\right)}$$

where $\rho_{0}=\rho \left(t=0\right)$ is the initial density of photoinduced excess charge carriers, as it can be easily verified by using t=0 and hence $\exp\left(-t/\tau \right)=1$ in the expression. Inserting then the expression (5) into Equation (2), we get the following expression for time-resolved photoluminescence decay:

(6) $$\phi \left(t\right)=\frac{BN\rho_{0}\exp\left(-t/\tau \right)}{1+b\rho_{0}\tau \left(1-\exp\left(-t/\tau \right)\right)}+\frac{B\rho_{0}^{2}\exp\left(-2t/\tau \right)}{{\left[1+b\rho_{0}\tau \left(1-\exp\left(-t/\tau \right)\right)\right]}^{2}}$$

The initial density of the excess charge $\rho_{0}$ is linked to the excitation fluence F by the equation $\rho_{0}=\alpha F/\hbar \omega$, which expresses the fact that one pair of free carriers (i.e., an electron and a hole) is generated for each photon of energy ℏω belonging to an excitation pulse of fluence F absorbed by the material. The 1:1 correspondence between photon density and charge carrier density assumes that multi-photon absorption processes are negligible in comparison with linear absorption. This is indeed correct in our case, in which supra-gap radiation is used to excite the material.

The peculiar characteristic of the function (6) is that for a long time $\left(t\gg \tau \right)$ it tends to a single-exponential function, and thus shows a constant and $\rho_{0}$-independent (i.e., fluence independent) logarithmic slope, while instead the initial temporal behavior significantly depends on the value of $\rho_{0}=\alpha F/\hbar \omega$, i.e., it depends on the laser fluence.

Extracting physically relevant information from the fitting of experimental data through fittings of experimental data using Equation (6), is complicated, due to the number of unknown quantities that appear in it. In this work, we propose as a useful procedure for data analysis, to extract an “instantaneous recombination rate” $W_{eff}\left(t\right)$, by means of the *logarithmic derivative* of $\phi \left(t\right)$, i.e.,:

(7) $$W_{eff}\left(t\right)≝-\frac{1}{\phi \left(t\right)}\cdot \frac{d\phi \left(t\right)}{dt}=-\frac{d\left(\ln\phi \left(t\right)\right)}{dt}$$

By integrating (6) we obtain $\phi \left(t\right)=\phi \left(0\right)\exp\left\{-\int_{0}^{t}w_{eff}\left(t^{\prime }\right)dt^{\prime }\right\}$, which shows again that the simple case of a time-independent instantaneous recombination rate corresponds to simple single-exponential (first order) kinetics.

At a generic instant, t, the instantaneous recombination rate describes (by definition) the logarithmic slope of the $\phi \left(t\right)$ curve, which is related to the laser fluence, as mentioned before. Therefore, we have a link between the instantaneous recombination rate at instant t=0 and the laser fluence; by simple algebra we can calculate the derivative in (6) at t=0 and obtain the following relationship:

(8) $$W_{eff}\left(t=0\right)=\left(\frac{1}{\tau }+b\rho_{0}\right)\left(\frac{N+2\rho_{0}}{N+\rho_{0}}\right)$$

By using the expression (8), it is possible to determine the initial recombination rate via a numerical derivative of the TRPL data. Next, the extraction of best-fit values of *b*, *τ* and *N* becomes possible by determining the *ρ*_0_ quantities (i.e., the densities of photo-injected excess carrier) at different optical fluencies. This procedure avoids performing more complicated fittings of Equation (6).

### 3.2. Experimental Results and Discussion

**Figure 1:**
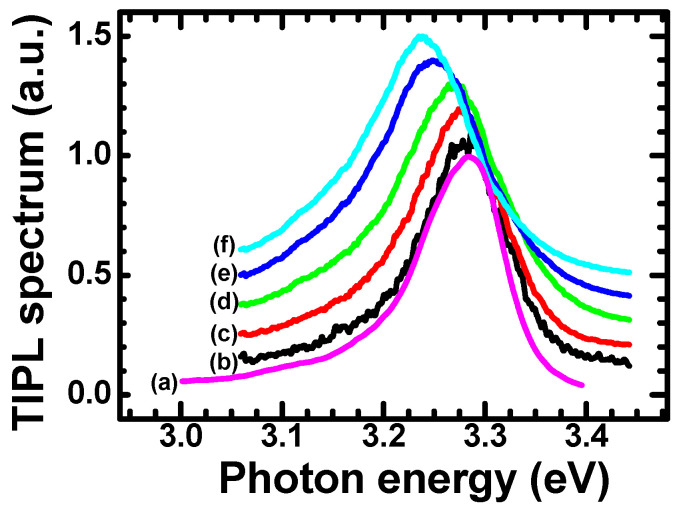
Time-integrated PL spectra measured at different laser fluencies. Curve (**a**): low-fluence CW excitation (HeCd laser). Curves from (**b**) to (**f**) are obtained using the pulsed laser excitation and at optical excitation fluencies of 10, 50, 276, 1190 and 1940 μJ cm^−2^ for curves from (**b**) to (**f**), respectively. Please note that the curves are vertically shifted to improve the figure readability.

Representative examples of a low-fluence CWPL spectrum and of TIPL spectra acquired for different values of laser fluence, are reported in Figure 1. The spectra refer to the ultraviolet range of the ZnO photoluminescence emission (excitonic emission).

As expected for room temperature measurements, spectral features related to bound excitons and donor–acceptor pair recombination [33] are not evidenced, while an unstructured emission spectrum that peaked at about 3.28 eV was observed in all samples.

The curve (a) was obtained using the HeCd laser as the excitation source. As the HeCd is a continuous-wave source, it distributes the energy over long time periods and leaves stationary electron-hole density much lower (about four orders of magnitude lesser) than the instantaneous charge carrier density, determined by pulsed laser excitation. The PL spectra measured via picosecond pulsed excitation for different values of *F* (laser fluencies) are reported in curves (b)–(f).

The TIPL spectra have been measured to assure that the optical fluences involved are weak enough to avoid the activation of the “p-line” (caused by exciton–exciton scattering), observable as a PL contribution peaking at 3.18 eV [34]. It is also possible to infer that the experimental conditions do not cause the dissociation of excitons due to the screening of the Coulomb potential by electron-hole plasma, which would lead to an emission peak at even lower photon energies [34,35], which are not observed here.

No significant shift of the PL peak was noticed for excitation fluencies of a few μJ/cm^2^ (see curve (b) of Figure 1), while a red shift of PL peak emission vs. laser fluency is evidenced, with values compatible with the bandgap narrowing caused by electron–electron and hole–hole exchange interactions [36]. The maximum measured PL shift was about 50 meV for the employed values of laser fluences. This value is small enough to ensure that that neither the activation of the p-line nor the plasma-induced exciton dissociation occurs. Hence, we can still model the measured PL in the frame of interband recombination.

**Figure 2:**
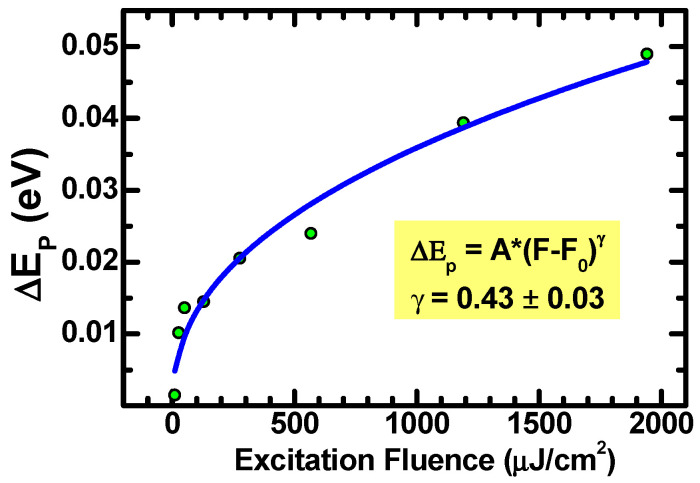
Shift of PL peak energy vs. excitation fluency F. The data are obtained by the measurements reported in Figure 1 and the “zero value” of the shift refers to the low fluence measurement obtained via the use of an HeCd laser. The blue curve is a best fit of the data obtained through the function in Equation (9).

The PL spectra of Figure 1 exhibit a small energy shift toward lower energies, but retain the same spectral widths and shapes at increasing excitation fluences. These facts suggest that the excitons retain their individual character and binding energy at investigated fluencies, while the shift of the emission peak results from the bandgap narrowing. This is supported by the evidence shown in Figure 2, where the shift $\Delta E_{p}$ of emission peak energy is plotted vs. the excitation laser fluency, *F*. Notably, a power law provides a good fitting:

(9) $$\Delta E_{p}\left(F\right)=A\cdot {\left(F-F_{0}\right)}^{\gamma }$$

where *F* and $\Delta E_{p}$ are reported in μJ/cm^2^ and in eV units, respectively. The same function as in Equation (9) has been employed by Schmid et al. [37] to describe the gap shrinkage in heavily n-doped and p-doped silicon versus doping concentration, while Lu and co-workers [36] showed that the same empirical power law behavior also fits with the experimental bandgap narrowing in aluminum-doped ZnO films. Notably, the value γ=0.43±0.03 found for the data in Figure 2 is very close to the one (γ=0.40) obtained and reported in the mentioned work [36].

As mentioned before, the linear absorption of the excitation laser pulses dictates the relationship $\rho_{0}=\alpha F/\hbar \omega$ between the initial density of photogenerated charges and the laser fluence *F*, where *α* is the absorption coefficient at the photon energy ℏω = 3.49 ev, corresponding to the laser wavelength of 355 nm.

**Figure 3:**
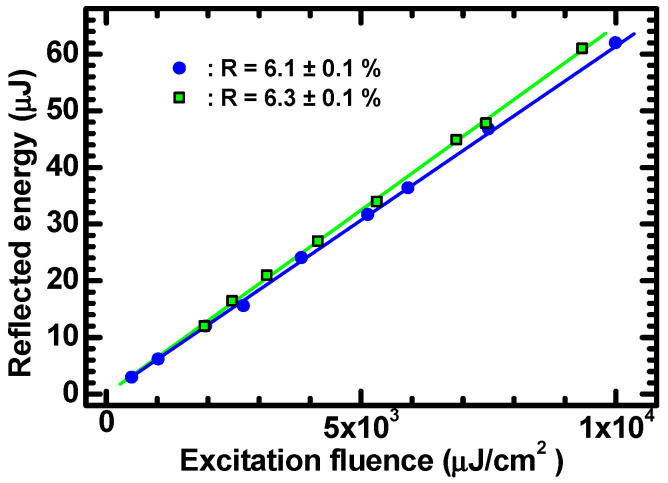
Reflected energy vs. excitation fluency at *λ* = 355 nm for two of the investigated ZnO crystals. The data refer to two ZnO crystals and indicate constant (i.e., linear) reflectivities of 6.1 ± 0.1% (sample 1, blue circles) and of 6.3 ± 0.1% (sample 2, green squares).

As mentioned before, the proportionality relationship between $\rho_{0}$ and F might be modified by nonlinear contributions to the optical absorption. In order to check for eventual nonlinear effects, measurements of crystal reflectivity at 355 nm were performed at various excitation fluencies. As shown in Figure 3, no fluence-dependent variation in reflectivity was observed in the investigated range. This indicates that it is indeed reasonable to neglect nonlinear optical effects and use the relationship $\rho_{0}=\alpha F/\hbar \omega$ to describe the experimental data.

A reference value of *α*(*λ* = 355 nm) = 1.5 · 10^5^ cm^−1^ was taken from optical transmittance analysis of ZnO single-crystal films, as reported by Muth et al. [38]. Consistent values for absorption coefficient were also reported in earlier optical transmission analysis of ZnO platelets [39] and in ellipsometry analysis of ZnO single crystals [40]. Using this reference value, densities of photogenerated charge carriers of 2 × 10^18^ cm^−3^, 5.5 × 10^19^ cm^−3^, 2.4 × 10^20^ cm^−3^ and 3.9 × 10^20^ cm^−3^ were obtained for curves from (b) to (f).

We would like to underline that, regardless of the value of the absorption coefficient, the red shift of the photoluminescence spectra becomes evident at an excitation fluency of ~100 μJ/cm^2^. This suggests probing optical excitation densities at such a value and above for the TRPL experiments.

**Figure 4:**
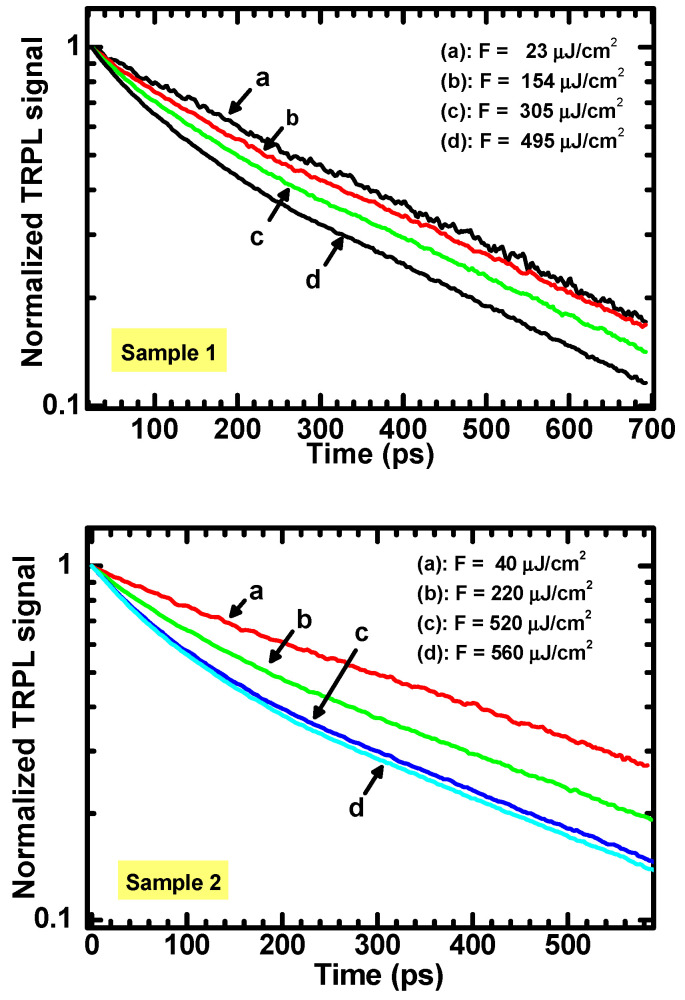
Peak-normalized TRPL curves measured at different values of excitation fluence.

Figure 4 shows some of the peak-normalized TRPL curves measured for the ZnO samples at different values of optical fluence. The reported PL signal is obtained by integration over the UV peak. The curves shown on Figure 4 are just a representative excerpt of the set of measurements performed. It is also to be underlined that the data reported in Figure 4 are different from the data used to determine the TIPL spectra of Figure 1, so that there is no correspondence between the optical fluencies in Figure 1 and Figure 4.

The data in Figure 4 evidence that different decay kinetics occur for different values of excitation fluence, and thus for different initial values of the charge densities. At larger $\rho_{0}$ values, the TRPL signal starts with a fast non-exponential decay, whose slope is fluence-dependent and which dominates for about the first 200 picoseconds. Successively, a slower decay is observed, characterized by an almost constant and laser fluency-independent logarithmic slope of the signal. Once the laser fluency is decreased, the fast component of TRPL becomes less evident, until the decay curve tends to a simple single-exponential.

**Figure 5:**
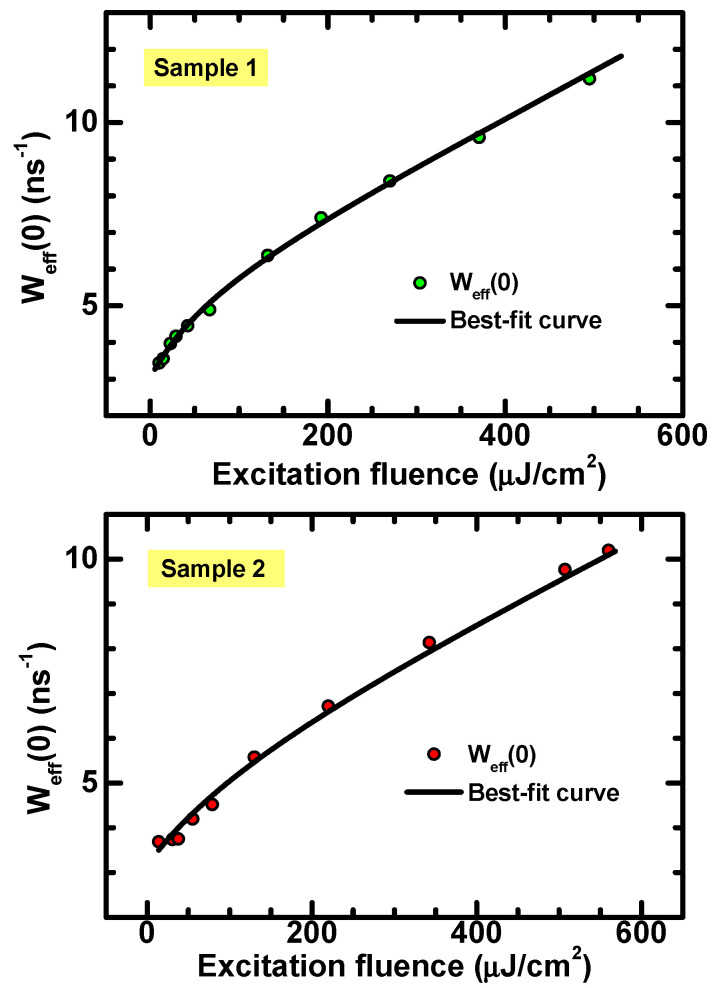
Instantaneous recombination rate calculated at t = 0 vs. excitation fluence. The black curves are the best fit of the data obtained by use of Equation (8).

As discussed previously, this behavior shows the characteristic features for a second-order recombination rate and agrees with the PL decay reported in Equation (6). Therefore, from decay curves acquired at different values of the laser fluence F, the instantaneous lifetime was determined via numerical derivative and the initial values were fitted as a function of *F* by using Equation (8). The results are reported in Figure 5.

The analysis results are consistent with the predictions, as they give a lower value for $W_{eff}\left(0\right)$, which is close to the fluence-independent slope (i.e., 1/τ) of the TRPL curves. On the other hand, $W_{eff}\left(0\right)$ increases at larger values of the laser fluence. The black curves represent the best fit of the data obtained through Equation (8) and using $\rho_{0}=\alpha F/\hbar \omega$. The values for the quantities *N*, *b* and *τ* obtained from best fits are reported in Table 1.

Additional considerations can be elaborated by considering the second order expression of the non-radiative rate of Shockley-Read-Hall recombination. For recombination centers whose energy levels are close to the center of the bandgap, this rate is given by [12]:

(10) $$W_{SRH}=\frac{\rho N+\rho^{2}}{\tau_{min}\left(N+\rho \right)+\tau_{maj}\rho }≅\frac{\rho }{\tau_{min}}-\frac{\rho^{2}\tau_{maj}}{N\tau_{min}^{2}}$$

where $\tau_{min}$ and $\tau_{maj}$ are the SRH lifetimes for the minority carrier and majority carrier, respectively [12]. In Equation (10), we also show the expansion of the SRH rate up to the second order in the density of photogenerated carriers, so that it is possible to estimate the radiative bimolecular coefficient *B* and the SRH non-radiative minority lifetime $\tau_{min}$. In fact, by neglecting third-order terms (i.e., Auger rate) in the total recombination rate $W^{\left(2\right)}=W_{R}+W_{SRH}$, and using Equations (3), (4) and (10), we get the relations $1/\tau =BN+1/\tau_{min}^{SRH}$ and $b=B-\tau_{maj}^{SRH}/N{\left(\tau_{min}^{SRH}\right)}^{2}$.

Rough but reasonable estimations can be made by considering similar values for the non-radiative lifetimes of the majority and minority carriers (i.e., $\tau_{min}^{SRH}≅\tau_{maj}^{SRH}$) [12], so that we obtain values of 0.8 ns for $\tau_{min}$ (for both samples) and values of B ≈ 1 × 10^−10^ cm^3^ s^−1^ (sample 1) and B ≈ 0.7 × 10^−10^ cm^3^ s^−1^ (sample 2) for the radiative bimolecular coefficient *B*, which are reasonably close to room temperature *B* values in epitaxial GaAs [41,42] and GaAs/AlGaAs quantum wells [43].

## 4. Conclusions

In conclusion, we proposed here a relatively simple method for the extraction of the parameters defining the second-order kinetics that rule the interband recombination in ZnO crystals. The goal of this work was to highlight the possibility of extracting well-defined (i.e., physically meaningful) parameters from TRPL data without resorting to phenomenological fittings. This aim is, to our opinion, especially important for semiconductors that exhibit significant light-emitting, photocatalytic or gas-sensing properties. In this sense, ZnO is a particularly suitable material, as it exhibits all these three functionalities.

The key point allowing the method described here is that, within a suitable range of laser excitation fluences, the interband recombination of ZnO crystals is very well described using second-order kinetics for the total recombination rate, characterized by an onset of non-exponential (bimolecular) decay observed for carrier densities of about 10^19^ cm^−3^. This allowed us to determine and fit the initial values of the “instantaneous” recombination rate (Equations (7) and (8)) measured at different excitation fluencies. The procedure allowed us to extract the parameters b and *τ* by simple data fittings and may represent a useful tool for quantitatively characterizing the charge recombination in ZnO.

Furthermore, we also proved that the procedure provides information on radiative vs. non-radiative recombination contributions by estimating the bimolecular radiative coefficient *B* and the non-radiative Shockley-Read-Hall lifetime, without the need to perform further experiments (e.g., measuring the quantum efficiency of PL emission).

We propose the procedure developed here as a tool for sketching a quantitative outline of the ZnO exciton recombination dynamics, going beyond the use of TRPL analysis as a mere characterization technique.

## Figures and Tables

**Table 1:** Recombination parameters and equilibrium free carrier density extracted by the data analysis.

	Sample 1	Sample 2
τ (ns)	0.33 ± 0.04	0.32 ± 0.04
b (cm^3^ s^−1^)	(3.0 ± 0.3) × 10^−11^	(2.2 ± 0.3) × 10^−11^
N (cm^−3^)	(1.3 ± 0.6) × 10^19^	(3 ± 1) × 10^19^

## Data Availability

All data are included in the paper.

## Appendix A

To improve the readability of the manuscript, some definitions and explanations are reported here in the Appendix.

*Unimolecular lifetime*, τ: This is the quantity often referred to as simply “lifetime”. It defines the linear (i.e., first order) term of a decay rate in a kinetic equation of the form −dρ/dt=ρ/τ. It is clear to see that the solution of this equation is a single-exponential. This kinetic can describe the recombination for very low optical injection levels, i.e., when the density of photogenerated carriers is low compared to the density of equilibrium carriers due to the doping. However, it cannot describe the recombination between two photogenerated carriers, which is a bimolecular recombination.

*Bimolecular coefficient*, *b*: This describes the probability of a recombination (either radiative or non-radiative) between a photogenerated hole and a photogenerated electron, according to a quadratic (two-body) rate equation of the form $-d\rho /dt=b\rho^{2}$, whose solution is clearly non-exponential.

*Radiative bimolecular coefficient*, *B*: This is the analogue of the b coefficient for the radiative recombination processes only, i.e., $Bnp=B\rho \left(\rho +N\right)$ is the number of photons produced via electron-hole radiative recombination per unit time and volume.

Once terms of third order and higher (i.e., $\rho^{3}$, $\rho^{4}$ etc.) are neglected, the number of recombination events (either radiative or non-radiative) per unit time and volume shall include a linear (“unimolecular”) term and a quadratic (“bimolecular”) term, which lead to the general expression of Equation (4). It is clear that for densities of optically-excited carriers that are negligible with respect to the equilibrium charge density N (the one caused by doping), the overall kinetic will be dominated only by the first term, i.e., a single-exponential PL decay shall be expected. For higher values of ρ, the deviation from single-exponential is expected to become more and more relevant.
